# A Broad-Host-Range Tailocin from Burkholderia cenocepacia

**DOI:** 10.1128/AEM.03414-16

**Published:** 2017-05-01

**Authors:** Guichun W. Yao, Iris Duarte, Tram T. Le, Lisa Carmody, John J. LiPuma, Ry Young, Carlos F. Gonzalez

**Affiliations:** a Department of Plant Pathology and Microbiology, Texas A&M University, College Station, Texas, USA; b Center for Phage Technology, Texas A&M University, College Station, Texas, USA; c Department of Pediatrics and Communicable Diseases, University of Michigan Medical School, Ann Arbor, Michigan, USA; d Department of Biochemistry and Biophysics, Texas A&M University, College Station, Texas, USA; McMaster University

**Keywords:** tailocin, Burkholderia cenocepacia, broad host range, LPS, receptor

## Abstract

The Burkholderia cepacia complex (Bcc) consists of 20 closely related Gram-negative bacterial species that are significant pathogens for persons with cystic fibrosis (CF). Some Bcc strains are highly transmissible and resistant to multiple antibiotics, making infection difficult to treat. A tailocin (phage tail-like bacteriocin), designated BceTMilo, with a broad host range against members of the Bcc, was identified in B. cenocepacia strain BC0425. Sixty-eight percent of Bcc representing 10 species and 90% of non-Bcc Burkholderia strains tested were sensitive to BceTMilo. BceTMilo also showed killing activity against Pseudomonas aeruginosa PAO1 and derivatives. Liquid chromatography-mass spectrometry analysis of the major BceTMilo proteins was used to identify a 23-kb tailocin locus in a draft BC0425 genome. The BceTMilo locus was syntenic and highly similar to a 24.6-kb region on chromosome 1 of B. cenocepacia J2315 (BCAL0081 to BCAL0107). A close relationship and synteny were observed between BceTMilo and Burkholderia phage KL3 and, by extension, with paradigm temperate myophage P2. Deletion mutants in the gene cluster encoding enzymes for biosynthesis of lipopolysaccharide (LPS) in the indicator strain B. cenocepacia K56-2 conferred resistance to BceTMilo. Analysis of the defined mutants in LPS biosynthetic genes indicated that an α-d-glucose residue in the core oligosaccharide is the receptor for BceTMilo.

**IMPORTANCE** BceTMilo, presented in this study, is a broad-host-range tailocin active against Burkholderia spp. As such, BceTMilo and related or modified tailocins have potential as bactericidal therapeutic agents against plant- and human-pathogenic Burkholderia.

## INTRODUCTION

The Burkholderia cepacia complex (Bcc) is a group of Gram-negative bacterial species, most of which are found in the natural environment and are not pathogenic to healthy humans. Some of these species, however, are opportunistic pathogens and pose serious risks to the health of persons with cystic fibrosis (CF) (1, 2). Among Bcc species, Burkholderia cenocepacia, especially the ET12 epidemic strain, is more commonly associated with cepacia syndrome, a rapidly progressing and usually fatal necrotizing pneumonia (3, 4). Most clinically relevant Bcc strains have demonstrated broad-spectrum antibiotic resistance *in vitro* (5). Even with a combination of antibiotics, clearance of infection is not generally observed (6). There is a substantial need to develop new strategies for antimicrobial therapy against this group of pathogens.

One potential strategy is the use of tailocins, or phage tail-like bacteriocins (7). These large bactericidal structures, first identified as R-type and F-type pyocins produced by Pseudomonas aeruginosa, resemble phage tails, with the R-type pyocins corresponding to the contractile tails of myophages such as T4 and the F-type pyocins corresponding to the flexible, noncontractile tails of siphophages such as T1. Similar bactericidal complexes were identified in many other bacterial genera (8–10). The term “tailocin” was recently coined both to highlight the similarity to phage tails and to avoid confusion with the small-molecule bacteriocins (11). By extension, tailocins with contractile tail and flexible tail morphologies are designated myotailocins and siphotailocins, respectively. Both types of tailocins reproduce the initial steps of a phage infection cycle. After specific adsorption to a phage receptor, there is a conformational change in the tail structure, resulting in the puncturing of the host cytoplasmic membrane, massive ion release, and collapse of the proton motive force. This conformational change is most dramatic in the myotailocin, with the tail sheath contracting, forcing the tail tube through the outer membrane of the host and leading to a puncturing of the inner membrane. In phage infections, ejection of the viral DNA into the cell is followed by resealing of the inner membrane lesion, thus allowing resumption of macromolecular synthesis and the progression of the infection cycle. In contrast, tailocins do not inject DNA, there is no resealing event, and the cells never recover from the puncturing event, resulting in single-hit, nonlytic lethality.

Studies performed in the early 1970s showed that Pseudomonas myotailocins could be effective as antimicrobial agents for treatment of peritonitis in mice (12, 13). However, R-type pyocins exhibit narrow bactericidal spectra (14) and the diversity of tail fibers observed in other systems has shown that tailocins in general have a narrow host range (10). Myotailocins were recently engineered (15, 16) to extend their host range by replacing the C-terminal domain of the tailocin tail fiber (11, 14).

Despite the extensive literature on bacteriocins, little has been reported about Burkholderia bacteriocins and tailocins. Gonzalez and Vidaver (17) first reported bacteriocin production from a small set of B. cepacia strains. In a larger study that included 373 isolates from plants and clinical or environmental sources, Govan and Harris (18) developed a typing system based on susceptibility and production of bacteriocins for B. cepacia and identified two myotailocins by electron microscopy (EM). More recently, a report addressing the ecological interaction of P. aeruginosa with members of the Bcc, which often coinfect patients with CF, noted that the majority of P. aeruginosa interspecific inhibitory activity was due to the production of myotailocins (19). We report here on the isolation and characterization of a broad-host-range tailocin from a strain of B. cenocepacia and the identification of its cell surface receptors.

## RESULTS

### Identification, induction, and morphology of tailocin BceTMilo.

In the process of screening for tailocins active against members of the Bcc, it was observed that B. cenocepacia BC0425 produced small clear zones of inhibition on overlays of 11/20 panel indicator isolates (see Table S1 in the supplemental material). Cultures of BC0425 induced with UV light underwent lysis and produced ∼5 × 10^10^ killing units (KU)/ml^−1^, indicating the presence of an inducible tailocin locus (20–22). Tailocins were purified and concentrated by ammonium sulfate precipitation followed by ultracentrifugation, achieving yields of ∼40% and final titers of ∼5 × 10^11^ KU ml^−1^.

**FIG 1:**
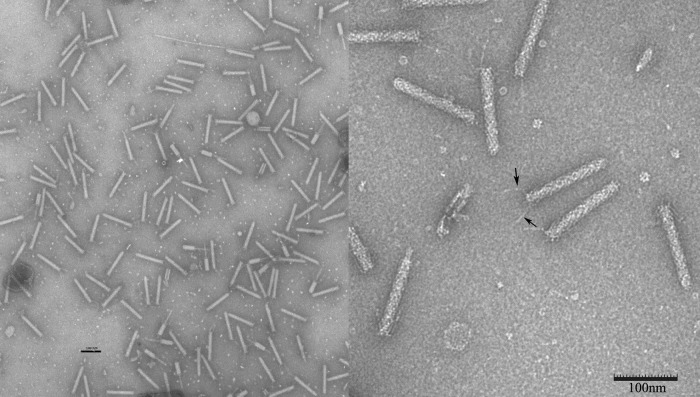
Transmission electron micrographs of IEF-purified tailocin BceTMilo. Samples were negatively stained with 2% (wt/vol) aqueous uranyl acetate. Bar, 100 nm. (Left panel) Magnification, ×25,000. Contracted tailocins are shown. (Right panel) Magnification, ×100,000. Arrows indicate tail fibers.

The tailocins, after further purification by preparative isoelectric focusing and visualization by transmission electron microscopy (TEM), displayed the characteristic morphology of R-type pyocins of P. aeruginosa (21). Electron micrographs of purified tailocin showed both uncontracted (∼87.4%) and contracted (∼12.6%) particles (Fig. 1, left panel). The uncontracted particles were rods that measured 141 ± 3.5 nm by 17 ± 1 nm, with a baseplate and several tail fibers visible in the electron micrograph (Fig. 1, right panel, arrows), and ∼33 to ∼34 rings of sheath proteins, as estimated by direct counting (not shown). Some particles were contracted, resulting in a shortening and thickening of the sheath (55 nm by 23 nm) and the exposure of a 140-nm-by-8-nm tail tube (Fig. 1, left panel). On the basis of the morphology, the myotailocin was designated BceTMilo, where “Bce” indicates the host (B. cepacia), “T” indicates that the item is a tailocin (rather than a phage), and Milo is the name assigned to the locus (on the basis of our convention that the names for phage and phage-derived particles with contractile, flexible, or short/stubby tails begin with M [myo], S [sipho], or P [podo], respectively) (https://cpt.tamu.edu).

### Characterization of the myotailocin BceTMilo.

Purified BceTMilo did not lose killing activity under conditions of exposure to trypsin, α-chymotrypsin, proteinase K, protease, lipase, papain, or lysozyme, consistent with other tailocins (22). BceTMilo killing activity was stable for 1 h at 50°C but was lost after 1 h at 80°C or after 3 min at 100°C; under conditions of storage at 4 to 8°C, the killing activity showed approximately 50% loss over a 6-month period. Tailocin killing activity was stable between pH 4.8 and pH 8.8, with a 100-fold decrease in activity after 18 h at pH 10.5 and all detectable activity lost after 18 h at pH 2.0.

**FIG 2:**
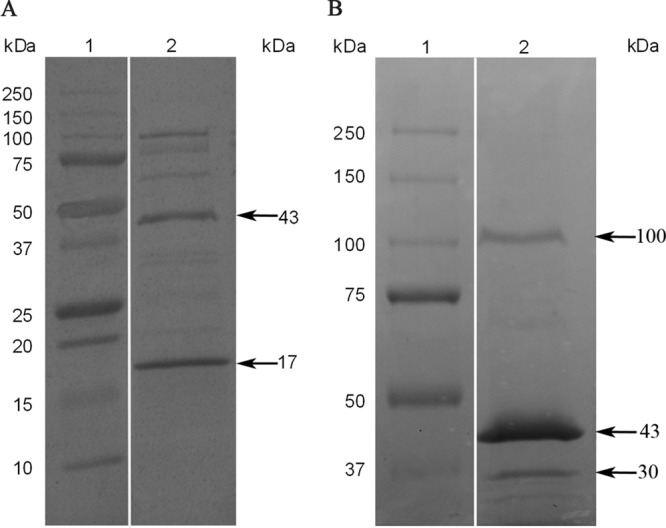
SDS-PAGE of IEF-purified tailocin BceTMilo. Protein subunits of tailocin BceTMilo were dissociated as described in Materials and Methods and loaded on a (A) 10 to 20% Tris-HCl SDS gel or (B) 7.5% Tris-HCl SDS gel. Lane 1, molecular mass standards (Precision Plus protein standard from Bio-Rad; 10 to 250 kDa); lane 2, subunits of tailocin BceTMilo. Proteins were visualized using Coomassie blue staining. Please note that the white lines between lanes 1 and 2 for both panel A and panel B indicate that a lane was removed from each of the original gels, since the data were not presented in Results.

SDS-PAGE analysis of isoelectric focusing (IEF)-purified tailocin revealed four major protein subunits of 17, 30, 43, and 100 kDa (Fig. 2). For myotailocins, which have structures similar to the tails of myophages such as T4, the two most numerous species should be the sheath and tube proteins, which typically have ∼40-kDa and ∼20-kDa molecular masses (23), corresponding to the 43-kDa and 17-kDa species (Fig. 2A). Given the 33 to 34 rings of sheath proteins as determined above, both sheath and tube proteins should be present at ∼200 molecules per tailocin particle. Densitometry analysis of the 43-kDa and 17-kDa tailocin protein species allowed quantification of tailocin particles and a calculation of their killing efficiency (Table 1). On the basis of the results, given the limitations of Coomassie blue staining, we estimated an average efficiency of killing (EOK) of ∼0.5 (Table 1). This strongly indicates that BecTMilo kills bacterial targets by the same single-hit killing mechanism as that characterized for other myotailocins (7). On the basis of this estimate, it is possible to calculate that induced cells produce on average approximately 620 ± 30 KU or particles cell^−1^ (Table 2).

**TABLE 1:** Tailocin BceTMilo efficiency of killing

Tail major protein	Protein mass (ng/10 μl)	No. of protein molecules^*a*^ (10^14^ per ml)	No. of tailocin particles^*b*^ (10^11^ per ml)	No. of KU^*c*^ (10^11^ per ml)	EOK^*d*^
Sheath (43 kDa)	91	1.3	6.27	3.89	0.6
	144	2.0	9.89	3.89	0.4
	151	2.1	10.40	12.30	1.2
	473	6.6	32.40	12.30	0.4
Tube (17 kDa)	36	1.3	6.20	3.89	0.6
	94	3.3	16.30	3.89	0.2
	322	11.0	55.80	12.30	0.2
Avg					0.5

a Number of protein molecules = [(protein mass × 1E−9) × 100/molecular mass] × Avogadro's number.

b Number of tailocin particles = number of protein molecules/204 (calculated as the number of protein molecules [tube or sheath]/the number of tailocin particles).

c KU, killing units.

d EOK = number of KU/number of tailocin particles.

**TABLE 2:** Burst size of tailocin BceTMilo^*a*^

Expt	Total no. of cells (10^9^)	Total no. of KU (10^12^)	No. of KU/cell
1	7.1	4.73	660
2	3.5	2.14	610
3	6.0	3.49	580
Avg			620

a Burst size was calculated as the total number of killing units (KU) of lysates divided by the total number of cells exposed to UV.

### Mapping and features of the BceTMilo defective prophage.

**FIG 3:**
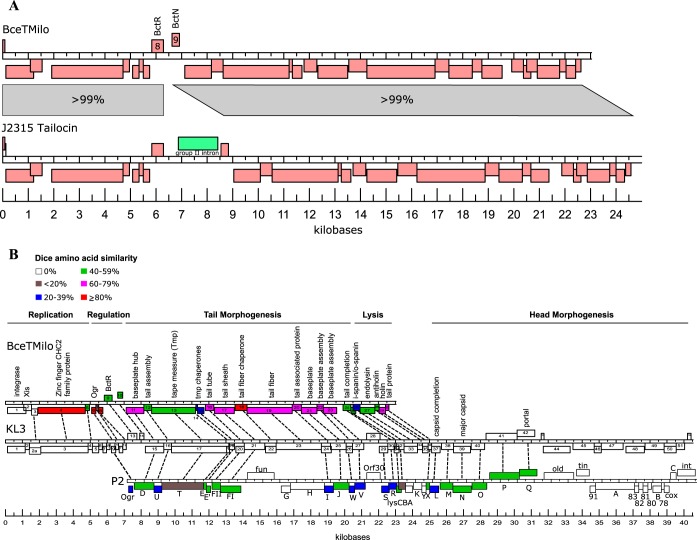
Genomic map of BceTMilo and syntenic comparison. (A) Syntenic relationship of tailocin BceTMilo to tailocin locus on B. cenocepacia J2315. (B) Syntenic comparison of tailocin BceTMilo, Burkholderia phage KL3, and Enterobacteria phage P2. The genomic maps were drawn to scale. Genes are color coded according to Dice amino acid similarity compared to Burkholderia phage KL3, and the related genes are indicated with dotted lines.

To determine the genetic locus encoding the tailocin, a draft genome of strain BC0425 was compared to peptide sequences obtained by liquid chromatography-tandem mass spectrometry (LC-MS/MS) analysis of the tail sheath and tube proteins as well as of the two minor proteins. All four proteins could be mapped to a 23-kb contig of the draft genome (Fig. 3A), which contained 30 predicted phage-related genes (Table S3). The same element was found in the fully annotated genome of B. cenocepacia J2315 (Fig. 3A) and in those of many other Bcc strains (see Fig. S1 in the supplemental material), with the J2315 locus sharing >99% sequence identity over most of the genes, except for a 1.9-kb insertion, identified as representing a group II intron, between genes 8 and 9 (Fig. 3A).

A syntenic arrangement of genes was also found in the Bcc myophage KL3 (24), a phage obtained as a spontaneous plaque former in a lawn of its own lysogen; KL3 is shown in its prophage form in Fig. 3B. The morphogenesis genes of KL3, and thus also of BceTMilo, are mostly syntenic with respect to the well-studied paradigm temperate myophage P2 of Escherichia coli (Fig. 3B), making it possible to assign the function of most of the tailocin genes. For example, BceTMilo genes 16 and 17, which encode the 17-kDa and 43-kDa major tailocin species, correspond to the tail tube and sheath genes of KL3 and P2. Also, the genes encoding the 100-kDa and 30-kDa minor species of the tailocin are syntenic with respect to the tail fiber and tail fiber chaperone genes of KL3 and occupy the same positions in the late gene transcript pattern as the same but unrelated genes in P2 (Fig. 3B). As expected, the BceTMilo locus lacks the distal end of the KL3 prophage containing the head morphogenesis and DNA modification genes. Remarkably, the breakpoint in synteny between the tailocin locus and the morphogenesis gene region of KL3 and P2 is between the most distal tail protein (tail completion protein X) and the first capsid protein (head completion protein L). The only tail morphogenesis gene present in KL3 and P2 but missing in the tailocin locus is the S gene, which was apparently deleted along with a moron element (25) encoding a DNA methylase inserted in the antisense orientation within the tail morphogenesis genes. In P2, the S protein appears to fulfill the role of an adaptor required to allow the completed tail to dock with the filled capsid (26), a function not required for a tailocin. Overall, all genes encoding the BceTMilo proteins involved in tailocin morphogenesis are also similar to the equivalent genes in the P. aeruginosa R-type pyocin locus, except for those encoding the tape measure and the tail fiber and the associated chaperones (not shown).

BceTMilo and KL3 also have very similar lysis genes, including those encoding a type I holin (*gp29* [i.e., product of gene 29]), a putative antiholin with four predicted transmembrane domains (TMDs) (*gp28*), and the two spanin subunits (*gp25* and *gp26* for the i-spanin and o-spanin subunit, respectively), as described by Lynch et al. (24). These lysis genes are unrelated to the P2 functional equivalents.

Combining synteny and sequence similarity, it was possible to assign to every BceTMilo gene a counterpart in KL3, except for genes 1 and 2. *gp1* encodes a tyrosine integrase (Int) with strong similarity to the P2 Int but was not detectably related to *gp1* of KL3, also an integrase of the same family. The overlap of gene 2 with the integrase gene and the fact that its product has winged-helix DNA binding motifs suggest that *gp2* encodes a Xis protein required for prophage excision. The only gene that cannot be robustly assigned a function is gene 3, which has homolog to a gene in a similar Burkholderia phage, phage BEK (GenBank accession no. CP008753). The corresponding open reading frame (ORF) in KL3 was not previously annotated and was named gene 2a (Fig. 3B).

BceTMilo shares its late gene regulation system with KL3 and P2, since all three encode the well-characterized Ogr transcription factor (27). The genes involved in lysogenic regulation of the KL3 prophage have not been precisely defined, but *gp9* of the BceTMilo tailocin locus was closely related to *gp13* of KL3 and encodes a CI-like repressor. Induction of BceTMilo was determined to be RecA dependent, as UV induction of the BC0425 Δ*recA* strain resulted in no production of the tailocin or lysis of cells in parallel experiments where the in-*trans* complement [strain BC0425 Δ*recA* (pMo168::*recA*-comp)] produced near-parental levels of tailocin as determined by the spot titer method (data not shown).

Comparison of BceTMilo with the tailocin locus in J2315 showed that, in addition to the 1.9-kb group II intron insertion between the two regulatory genes for the J2315 tailocin, there was a 36-bp insertion in the J2315 version of the tail fiber assembly gene, resulting in the expansion of Ser-Glu-Pro motif repeats already present in BceTMilo; this motif expansion lies in a position expected to influence host range. Moreover, there were a total of 121 single nucleotide polymorphisms (SNPs) over the 23-kb sequence. Most of the SNPs were shown in gaps between genes. All of the SNPs in genes preserved the reading frame; i.e., all either were silent or were missense changes. Of 30 missense SNPs, only 3 were detected in hypothetical genes of unknown function; others were in structural genes or in lysis cassettes. For example, there were 7 SNPs detected in the tail fiber gene and 8 SNPs in the tape measure protein, which represented 5.2 kb of the locus; and 2 and 3 SNPs were found in the i-spanin/o-spanin and endolysin genes, respectively. Tailocin activity has not been reported for J2315, which contains the fully functional BcepMu prophage (28). However, negative-stain electron microscopy of concentrated lysates obtained by UV induction of J2315 revealed low numbers of tail-like structures indistinguishable from those of the BceTMilo particles for which we were unable to find a sensitive host among 41 Bcc strains tested. Nevertheless, the stringent conservation of all the structural genes suggests that the tailocin encoded in J2315 may contribute to fitness and thus may be functional but with a different host range dictated by the repeat expansion in the tail fiber assembly gene.

### Inhibitory spectrum of tailocin BceTMilo.

To determine the inhibitory spectrum of the tailocin, a panel of 101 Burkholderia species was tested for sensitivity, using both broth microdilution and spot assays. Of the 73 isolates representative of the species belonging to the Bcc, 50 were sensitive to BceTMilo (Table S2), with 59% of B. cenocepacia isolates tested exhibiting sensitivity. The non-Bcc Burkholderia species assayed, B. gladioli and B. glumae, showed high sensitivity (93%; Table S2). Overall, 76% of Burkholderia species tested were sensitive to tailocin BceTMilo. Burkholderia isolates from a subset of the same panel were spot tested for susceptibility to pyocin R1, R2, and R5; only 0/47, 1/47, and 4/47 were sensitive to each of the respective pyocins (Table S2).

### LPS determinants as receptors for BceTMilo.

**FIG 4:**
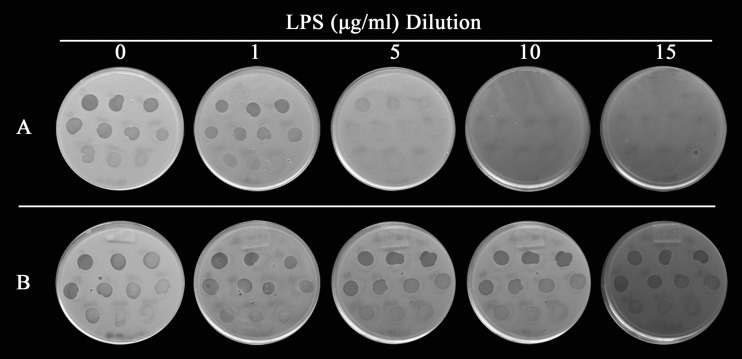
Effect of Burkholderia LPS on BceTMilo. Increasing amounts of purified LPS from (A) B. cenocepacia strain PC184 (sensitive) and (B) B. cenocepacia strain AU10487 (resistant) were mixed with tailocin BceTMilo and allowed to incubate for 30 min. A 1:2 dilution series (see Materials and Methods) of mixtures from the experiments represented in panel A and panel B were spotted onto overlays seeded with strain PC184 to determine residual BceTMilo activity. Results are representative of triplicate experiments.

To determine the nature of the receptor for tailocin BceTMilo, lipopolysaccharide (LPS) adsorption assays were conducted. LPS from a sensitive strain (PC184) and a resistant strain (AU10487) was extracted and used for the assay. Tailocin activity decreased in mixtures with increasing concentrations of LPS from the sensitive strain (Fig. 4A), whereas no decrease in activity was observed in mixtures with increasing concentrations of LPS from the resistant strain (Fig. 4B), indicating that the receptor for BceTMilo was part of the LPS of the sensitive strain.

**FIG 5:**
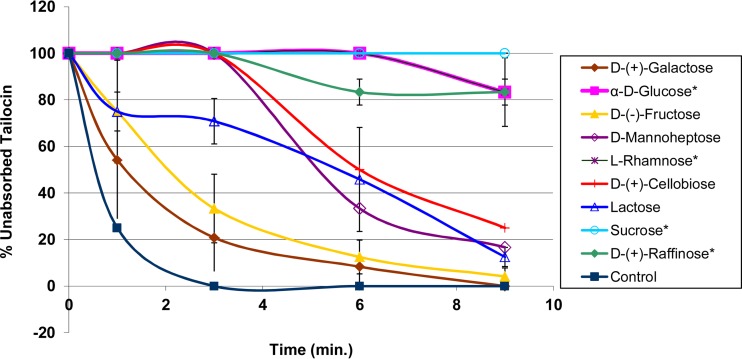
Sugar inhibition assay. Data represent the effects of d-galactose, α-d-glucose, d-fructose, d-mannoheptose, l-rhamnose, d-cellobiose, lactose, sucrose, and d-raffinose on tailocin adsorption to B. cenocepacia strain PC184. The titer was expressed as the reciprocal of the highest dilution showing inhibition. Asterisks (*) indicate sugars that inhibited tailocin BceTMilo adsorption. Results are representative of triplicate experiments. Each bar represents the standard deviation.

Since a sugar moiety(ies) of LPS could be involved in adsorption of the tailocin of BceTMilo, a sugar inhibition assay was conducted. Sugars α-d-glucose, l-rhamnose, and d-manno-heptose were chosen for evaluation since they are components of B. cenocepacia LPS (29, 30). Sugars α-d-glucose and α-l-rhamnose inhibited tailocin adsorption, whereas d-galactose and d-fructose had little or no effect on tailocin adsorption to sensitive cells (Fig. 5). The effects of di- and trisaccharides containing glucose were also investigated. Sucrose and raffinose, both with a α-1,2 glycosidic linkage, inhibited adsorption, whereas d-cellobiose and lactose, both with a β-1, 4 glycosidic linkage, showed only minor inhibition (Fig. 5).

**FIG 6:**
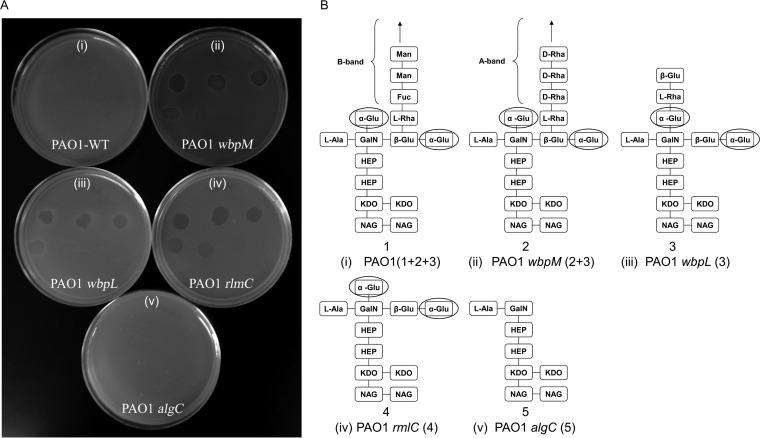
Sensitivity of P. aeruginosa LPS mutants to tailocin BceTMilo. (A) Activity of serially diluted (1:10) tailocin BceTMilo against PAO1 (i), PAO1*wbpM* (ii), PAO1*wbpL* (iii), PAO1*rmlC* (iv), or PAO1*algC* (v). WT, wild type. (B) Numbers in parentheses indicated in panel A correspond to depicted LPS structures with the same numbers as those indicated in panel B. Putative residues involved in BceTMilo adsorption are circled. Abbreviations: Man, mannose; Fuc, fucose; GalN, N-galactosamine; HEP, mannoheptose; NAG, *N*-acetylglucosamine; KDO, 2-keto-3 deoxyoctulosonic acid. (Adapted from reference 31 with permission of the publisher.)

Köhler et al. (31) have proposed that LPS plays an essential role both as a protective shield and as a receptor for R-pyocins of P. aeruginosa. Using genetically defined LPS mutants, they showed that the l-rhamnose residue was the receptor for R1 in P. aeruginosa and that two distinct d-glucose residues of the outer core were part of the receptor sites for R2- and R5-pyocins. We demonstrated here that P. aeruginosa strain PAO1 exhibited slight sensitivity to tailocin BceTMilo (Fig. 6A, panel i, and 6B, panel i; zones not clearly visible), whereas the PAO1 *wbpM* mutant derivative, deficient in the B-band, showed increased sensitivity to tailocin BceTMilo (Fig. 6A, panel ii, and 6B, panel ii). The *wbpL* derivative of PAO1, deficient in both the LPS B-band and A-band, and a PAO1 *rmlC* strain, devoid of l-rhamnose but containing an α-glucose residue, were both sensitive to BceTMilo (Fig. 6A, panel iii, 6B, panel iii, 6A, panel iv, and 6B, panel iv). However, an *algC* mutant, devoid of both l-rhamnose and α-d-glucose residues, exhibited no sensitivity to BceTMilo (Fig. 6A, panel v, and 6B, panel v), indicating that α-d-glucose is required for adsorption of tailocin BceTMilo in P. aeruginosa.

**FIG 7:**
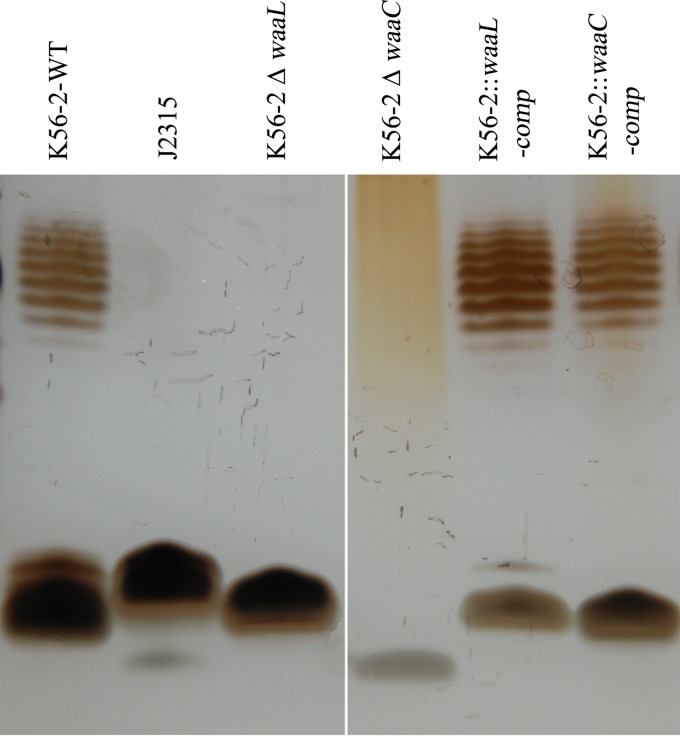
Electrophoretic profiles of LPS from B. cenocepacia K56-2, J2315, K56-2, and K56-2 deletion mutants and complements. LPS was prepared as described in Materials and Methods. A total of 1.0 μg LPS was loaded in each lane of a 16.5% polyacrylamide gel in a Tricine-SDS system and developed by silver staining as described in Materials and Methods. Please note that the white line between lanes 3 and 4 indicates that a lane was removed from the original gel, since the data were not presented for the strain in Results.

**FIG 8:**
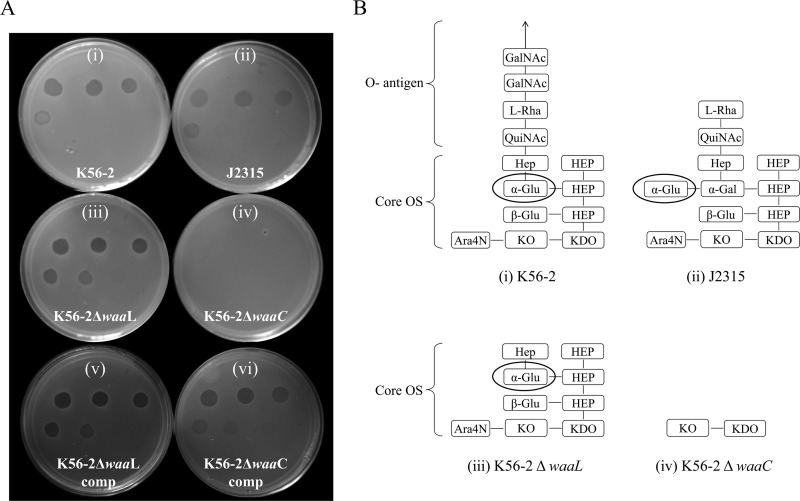
Sensitivity of B. cenocepacia strains K56-2 and J2315 and K56-2 LPS mutants to tailocin BceTMilo. (A) Activity of serially diluted (1:10) tailocin BceTMilo against strain K56-2 (i), J2315 (ii), K56-2 Δ*waaL* (iii), K56-2 Δ*waaC* (iv), K56-2 Δ*waaL* complement (v), or K56-2 Δ*waaC* complement (vi). (B) Numbers in parentheses indicated in panel A correspond to depicted LPS structure with the same numbers as those indicated in panel B. Residues involved in BceTMilo adsorption are circled. Abbreviations: OS, oligosaccharide; HEP, l-glycero-d-*manno*-heptose; KO, d-glycero-α-d-talo-oct-2-ulopyranolsylonic acid; KDO, 2-keto-3 deoxyoctulosonic acid; QuiNAc, β-d-QuiNAc. The structure shown in panel B was modified from Ortega et al. (30).

LPS mutants of genetically malleable B. cenocepacia strain K56-2 and their complements were constructed in this study, and their susceptibilities to BceTMilo were tested. Samples of LPS extracted from strain K56-2, strain J2315, or K56-2 deletion mutants and their complements were compared using SDS gel electrophoresis. Deletion of the *waaL* gene, encoding an O-antigen ligase, predicted a loss of O-antigen surface expression and the l-rhamnose in K56-2 (30), and deletion of *waaC*, a heptosyltransferase I, was predicted to result in a heptoseless mutant with a rough phenotype. A ladder-like banding pattern, corresponding to the presence of polymeric O-antigen, was visible for strain K56-2 (Fig. 7, lane 1), whereas J2315 was devoid of an O-antigen (Fig. 7, lane 2) as previously reported by Ortega et al. (32). The absence of an O-antigen ladder was observed in the mutant K56-2 Δ*waaL* preparation (Fig. 7, lane 3), and a severely truncated lipid A-core region with the concomitant loss of O-antigen production was observed in the K56-2 Δ*waaC* mutant (Fig. 7, lane 4). The in-*trans* complementation of the K56-2 Δ*waaL* mutant restored O-antigen production (Fig. 7, lane 5), and in-*trans* complementation of the K56-2 Δ*waaC* mutant restored O-antigen production and lipid-A core regions (Fig. 7, lane 6). Using the spot assay, we demonstrated that strains K56-2 and J2315 and the K56-2 *waaL* deletion mutant (Δ*waaL*) were sensitive to tailocin BceTMilo (Fig. 8A, panels i to iii) but that the Δ*waaC* mutant of K56-2 was not (Fig. 8A, panel iv, and 8B, panel iv). The complementation of the K56-2 Δ*waaL* deletion did not change the sensitivity to BceTMilo (Fig. 8A, panel v), but the complementation of the K56-2 Δ*waaC* deletion restored the sensitivity to BceTMilo (Fig. 8A, panel vi).

## DISCUSSION

Members of the Bcc and P. aeruginosa are significant opportunistic human pathogens in CF patients (33). Early lung infections in CF patients are most often due to P. aeruginosa; however, secondary infections caused by Bcc often give rise to highly variable and unpredictable clinical outcomes (34). Previous studies have suggested that P. aeruginosa and Bcc actively affect each other's growth through production of secreted products (35). Several studies have been reported that most clinical and environmental P. aeruginosa strains produce pyocins (19, 36, 37), whereas there is only limited information concerning bacteriocins produced by Burkholderia species (16, 17, 19). Bakkal et al. (19) noted that almost all P. aeruginosa isolates in their study were able to inhibit a wide range of P. aeruginosa and Bcc strains, whereas Bcc strains, on average, inhibited a much more limited number of strains.

In this study, we identified and characterized the tailocin BceTMilo, which exhibited broad host activity among Burkholderia spp. Evaluation of BceTMilo showed that 68% of Bcc (representing 10 species) and 90% of non-Bcc Burkholderia strains tested were sensitive (see Table S2 in the supplemental material). Additionally, tailocin BceTMilo had killing activity against P. aeruginosa PAO1 and derivatives.

The observed average of 600 particles per cell of BceTMilo produced by B. cenocepacia strain BC0425 is 3- to 6-fold higher than the 100 to 200 particles/cell reported for production of pyocin R2 by strain PAO1 (7). In P. aeruginosa, RecA, PrtR (repressor protein), and PrtN (activator protein) coregulate expression of the R-, F-, and S-type pyocin genes that are induced in responses to such mutagenic agents as UV and mitomycin C (38) or oxidative stress (39). Genome-wide transcriptome analysis of P. aeruginosa has revealed that pyocin S-, F-, and R-type transcripts are upregulated by oxidative stress, as are lysis-related genes, indicating that multiple pyocins are released upon induction (39). This coordinated transcription, translation, and lysis of cells may limit the number of R-type pyocin particles produced per cell.

The synteny and close relationship between BceTMilo and KL3 and, by extension, the paradigm temperate myophage P2 suggest that, other than regulatory genes, nothing is needed to generate a tailocin other than the deletion of the head genes (Fig. 3B). Since every structural gene required for the P2 myophage tail has a homolog both in KL3 and in the tailocin locus, the simplest explanation could be that both the BceTMilo myotailocin locus and the temperate myophage KL3 phage were derived from a common ancestor, with BceTMilo undergoing internal deletions of the head and DNA modification genes.

Comparison of BceTMilo with a tailocin locus in epidemic strain J2315 showed more than 99% sequence identity in the genes, with the exceptions of three areas. There was a 1.9-kb group II intron insertion between the two regulatory genes for J2315 tailocin, there was a 36-bp insertion in the J2315 version of the tail fiber assembly gene, and there were a total of 121 SNPs over the 23-kb sequence. The group II intron insertion between the two regulatory genes may have affected transcription efficiency and may have resulted in the observed low production of particles from J2315. The observed SNPs were dispersed throughout the J2315 tailocin cassette and did not result in frameshifts that would affect the levels of protein expression. As might be expected, the largest observed difference between BceTMilo and J2315 tailocin was in the tail fiber assembly gene, which determines the specificity of the two tailocins and therefore their host range spectra.

A critical feature for pyocins/tailocins is their ability to recognize and bind cell receptors specific to their host. Different bacterial surface components act as the receptors for phages and phage tail-like bacteriocins, including flagella (40), pili (41), outer membrane proteins such as OmpA and OmpC (42), and LPS (31, 43). LPS adsorprion assay results showed that the activity of tailocin BceTMilo was neutralized by the presence of purified LPS from sensitive strains but not by the presence of that from resistant strains (Fig. 4). Sugar inhibition assays indicated that l-rhamnose and sugars in which d-glucose was α-linked to another residue inhibited adsorption (e.g., sucrose or raffinose), whereas sugars with a β-linked d-glucose, as in d-cellobiose or lactose, did not inhibit adsorption (Fig. 5). This suggested that both α-d-glucose and l-rhamnose were the putative sugar components of the LPS involved in BceTMilo adsorption.

Our evaluation of BceTMilo for activity against PAO1 and LPS mutant derivatives showed that the parental strain, expressing both the A-band and B-band O polysaccharides, was less sensitive than strains PAO1 *wbpM* (LPS A^+^ LPS B^−^) and PAO1 *wbpL* (LPS A^−^ LPS B^−^), indicating that removal of the B-band LPS exposed the receptor site for the BceTMilo. The PAO1 *rmlC* mutant lacking the LPS A and B bands and a terminal l-rhamnose but containing a α-d-glucose residue was sensitive to BceTMilo, whereas the PAO1 *algC* mutant, devoid of both bands as well as of the terminal l-rhamnose and α-d-glucose residues, was resistant, suggesting that α-d-glucose is a receptor for adsorption of tailocin BceTMilo in P. aeruginosa.

The results obtained with PAO1 were correlated with the sensitivity patterns observed in B. cenocepacia J2315 and K56-2 and its derivative LPS mutants (Fig. 8). J2315 contains a terminal α-d-glucose and l-rhamnose and showed sensitivity to BceTMilo, as did K56-2, which contains an internal α-d-glucose in the core oligosaccharide and l-rhamnose as part of the O-antigen. The K56-2 Δ*waaL* (O-antigen-deficient) mutant devoid of l-rhamnose exhibited sensitivity to BceTMilo, which argues against the role of l-rhamnose as a receptor, despite the sugar inhibition data. Furthermore, the K56-2 Δ*waaC* (O-antigen- and α-d-glucose-deficient) mutant lacking the internal α-d-glucose was resistant to BceTMilo, which strongly suggests that α-d-glucose (in either an internal or a terminal position) is the receptor for the tailocin in K56-2 or J2315.

The host range activity of tailocins is determined by their tail fibers and generally exhibits a narrow spectrum of killing (14, 16). Tailocin BceTMilo exhibited broad host activity among Burkholderia species and lineages (Table S2). Bakkal et al. (19) noted that almost all P. aeruginosa isolates in their study were able to inhibit a wide range of P. aeruginosa and Bcc strains, whereas Bcc strains, on average, inhibited a much more limited number of strains, suggesting that bacteriocin-based inhibition may play a role in governing interactions of P. aeruginosa and Bcc in the CF lung. Results from our testing of 44 Bcc isolates, representing nine species, using pyocin R1, R2, or R5 lysates indicated very limited activity of the R-pyocins with respect to the Bcc (Table S2). Given that R2 encompasses the spectra of R3 and R4 (15, 44), we submit that it is possible that not all of the interspecific activity observed against the Bcc by Bakkal et al. (19) was due to pyocin activity and that the activity might have been a reflection of the isolates used in the study. Clearly, more information is needed to determine the role that tailocins play in the dynamics of microbial interactions.

Tailocins have previously been recognized as potential antibacterial agents (7, 45). The disadvantage of tailocins is obviously the incapacity for proliferation at the site of therapeutic action, thus requiring delivery at a high concentration. However, this disadvantage is somewhat compensated for by two fundamental aspects of the characteristics of tailocins: their lack of DNA (thus ameliorating concerns related to the unintentional spread of genes through specialized or generalized transduction or the dispersion of recombinant genetic material) and their one-hit killing character (which allows the definition of dosages in pharmacological usage). A further, somewhat less obvious limitation has been the rather narrow host range of the well-described tailocins, which may be related to the fact that hosts carrying tailocin loci cannot rely on DNA-based immunity for protection against the homotypic tailocin. Here, we have described a broad-host-range tailocin with activity against both human- and plant-pathogenic members of the genus Burkholderia. Recent studies performed with modified R-type tailocins have shown therapeutic promise in a murine model of P. aeruginosa peritonitis (7) and in Clostridium difficile infection (45). Evaluations of BceTMilo in both animal and plant models are needed to determine its potential as a treatment for Burkholderia infections.

## MATERIALS AND METHODS

### Bacterial isolates and culture conditions.

Bacterial strains and plasmids used in this study are listed in Table 3. The panel of 20 Burkholderia isolates included 18 B. cenocepacia isolates (7 members of the ET-12 lineage, 3 of the Mid-West clone, and 8 of the PHDC clone [2]) and 1 each of B. cepacia and Ralstonia pickettii (see Table S1 in the supplemental material). The 73 Bcc isolates and the three B. gladioli isolates used in sensitivity testing were stock cultures from the U.S. Burkholderia cepacia Research Laboratory and Repository (Ann Arbor, MI; Table S2). Burkholderia glumae isolates used in the study were obtained from rice field samples, collected in Texas, expressing bacterial panicle blight symptoms (C. F. Gonzalez, unpublished data) (Table S2). Tryptone nutrient broth (TNB) (52, 53) was used for liquid culture. Tryptone nutrient agar (TNA) used for routine maintenance of cultures was identical to TNB except that it lacked KNO_3_ and was supplemented with 20 g liter^–1^ agar. TNA soft agar (TNSA) (TNA with 7.5 g liter^−1^ agar) was used for overlays. Luria-Bertani (LB) medium was used in mating experiments (54). For counterselection, cointegrants were grown in yeast extract-tryptone (YT) broth, which consisted of 10 g liter^–1^ of tryptone and 10 g liter^–1^ of yeast extract in deionized water. Sucrose was added to YT broth at a final concentration of 15% (wt/vol) and was supplemented with 20 g liter^–1^ yeast extract-tryptone agar (YTA) to resolve cointegrants. All Burkholderia, E. coli, and P. aeruginosa strains were grown at 37°C in both liquid and solid medium unless stated otherwise. Antibiotics were added to the media at the following concentrations: 600 μg ml^–1^ kanamycin (Km) and 20 μg ml^–1^ tetracycline (Tc) for B. cenocepacia and 30 μg ml^–1^ Km for E. coli.

**TABLE 3:** Bacterial strains and plasmids used in this study

Strain or plasmid	Relevant characteristic(s)^*a*^	Reference or source
Strains		
Burkholderia cenocepacia		
Burkholderia cenocepacia BC0425	Producer of BceTMilo, ET12 epidemic lineage, clinical isolate	Laboratory collection
Burkholderia cenocepacia AU10487	Clinical isolate	Laboratory collection
Burkholderia cenocepacia K56-2	ET12 epidemic lineage, O-antigen^+^	Sokol et al. (46)
Burkholderia cenocepacia J2315	ET12 epidemic lineage, O-antigen^−^	Laboratory collection
Burkholderia cenocepacia PC184	Midwest epidemic lineage, O-antigen^−^	Laboratory collection
Burkholderia cenocepacia BC0425 Δ*recA*	BC0425, deleted *recA* gene	This study
Burkholderia cenocepacia K56-2 Δ *waaL*	K56-2, deleted *waaL* gene, O-antigen-l-Rham^−^	This study
Burkholderia cenocepacia K56-2Δ *waaC*	K56-2, deleted *waaC* gene, O-antigen-l-Rham^−^ Glu^−^	This study
Escherichia coli E. cloni 5-alpha	*fhuA2*Δ(*argF-lacZ*)*U169 phoA glnV44* Φ80Δ(*lacZ*)M15 *gyrA96 recA1 relA1 endA1 thi-1 hsdR17*	Lucigen
Pseudomonas aeruginosa PAO1	LPS A^+^ LPS B^+^, R2 pyocin producer	J. S. Lam (Burrows et al. [47])
Pseudomonas aeruginosa PAO1*wbpM*	LPS A^+^ LPS B^−^	J. S. Lam (Burrows et al. [47])
Pseudomonas aeruginosa PAO1*wbpL*	LPS A^−^ LPS B^−^	J. S. Lam (Rocchetta et al. [48])
Pseudomonas aeruginosa PAO1*rmlC*	LPS A^−^ LPS B^−^ l-Rham^−^	J. S. Lam (Rahim et al. [49])
Pseudomonas aeruginosa PAO1*algC*	LPS A^−^ LPS B^−^ l-Rham^−^ Glu^−^	J. S. Lam (Jarrell et al. [50])
Pseudomonas aeruginosa NIH-K	R1 pyocin producer	ATCC 25350
Pseudomonas aeruginosa NIH-1	R5 pyocin producer	ATCC 25313
Plasmids		
pRK2013	Tra^+^ Mob^+^ ColE1 replicon Km^r^	Laboratory Stock
pMo130	Suicide vector for allelic exchange in Burkholderia; ColE1 *ori* RK2 *oriT xylE sacB* Km^r^	M. I. Voskuil (51)
pMo168	Replicative vector for Burkholderia; *ori*pBBR1 *mob*^+^ *xylE* Km^r^	M. I. Voskuil (51)
pMo130::*recA*-UD	pM0130 with *recA* upstream and downstream fragments	This study
pMo130::*waaL*-UD	pM0130 with *waaL* upstream and downstream fragments	This study
pmo130::*waaC*-UD	pM0130 with *waaC* upstream and downstream fragments	This study
pMo168::*recA*-comp	*recA* gene cloned into pMo168	This study
pMo168::*waaL*-comp	*waaL* gene cloned into pMo168	This study
pMo168::*waaC*-comp	*waaC* gene cloned into pMo168	This study

a Km^r^, resistance to kanamycin; Rham, rhamnose; Glu, glucose.

### Tailocin induction study.

For a typical small-batch UV induction, strain BC0425 was grown on a TNA plate overnight at 37°C. A suspension was made and used to inoculate 20 ml TNB (*A*_600_ = 0.08), and the culture was grown with shaking (180 rpm) at 37°C to *A*_600_ = 0.5. The cells were pelleted by centrifugation (10,000 × *g* for15 min at 5°C), washed once with 0.85% (wt/vol) NaCl, repelleted, and resuspended in 10 ml of 0.85% (wt/vol) NaCl. The suspension was poured into a sterile 100-mm-diameter-by-15-mm-deep petri dish and exposed with the lid off to UV (360 μw cm^−2 s−1^) for 7 s in a class II biosafety hood. A 10-ml volume of 2× TNB and 30 ml of TNB were added to the treated and untreated control cells to make the final volume 50 ml. The cultures were returned to 37°C with shaking at 150 rpm. Growth of the culture was monitored using a Nephelo culture flask. When the *A*_600_ level decreased to below 0.2 for the UV-treated culture, DNase I was added to make a final concentration of 2 U ml^–1^ and the cultures were shaken at 37°C for an additional 30 min. Cell debris was removed by centrifugation (17,418 × *g* for 30 min at 5°C) followed by filtration through a 0.22-μm-pore-size filter (Millipore, Billerica, MA). Production of pyocins R1, R2, and R5 for spot sensitivity testing was accomplished by UV induction as described above. Titers of the lysates were determined as described below using strain PC184 and PAO1 *wbpM* as the indicators for tailocin BceTMilo and R-pyocins, respectively. To calculate the burst size, the CFU was determined prior to UV exposure and the number of tailocin particles produced was estimated based on a Poisson distribution killing assay, as described below. The tailocin burst size was calculated as the total number of killing units (KU) divided by the number of cells at the time of UV induction.

### Tailocin activity assay.

The antimicrobial activity of the tailocin was determined semiquantitatively by a spot dilution method. Briefly, serial dilutions (2-fold, 5-fold, or 10-fold) of tailocin preparations were prepared in P-buffer (100 mM NaCl, 8 mM MgSO_4_, 50 mM Tris-HCl, pH 7.5) and 10 μl of each dilution was spotted onto TNA plates that had been overlaid with TNSA seeded with approximately 10^7^ CFU ml^–1^ of an indicator bacterium. After incubation for 18 h at 37°C, the plates were evaluated for zones of inhibition. The reciprocal of the highest dilution that formed a visible inhibition zone was defined as the value of relative activity in arbitrary units (AU).

Quantitative tailocin activity assays were performed using a Poisson distribution killing method, adapted from Williams et al. (14) and originally described by Kageyama et al. (55). Briefly, tailocin serial dilutions in P-buffer were added to 10^9^ CFU ml^−1^ target bacteria in TNB and incubated for 40 min at 37°C in triplicate. The samples were then serially diluted and plated on TNA to count surviving bacteria. The number of tailocin particles is related to the fraction of bacterial survivors in a Poisson distribution and is calculated as *m* = −ln(*S*), where *m* is the possible average number of lethal events (i.e., representing adsorbed tailocin particles) per bacterial cell and *S* is the fraction of survivors. The total number of active particles per milliliter (*m* × the number of cells per milliliter) was defined as the number of KU per milliliter on the basis of the assumption that adsorption in each sample was quantitative within the 40-min incubation period.

### Large-scale tailocin production and purification.

The method of Scholl and Martin (7) was used for large-scale preparations. Briefly, ammonium sulfate was slowly added to the filter-sterilized tailocin lysate to make a final concentration of 40% (wt/vol) with stirring on ice. After 18 h at 5°C, the ammonium sulfate precipitate was pelleted by centrifugation at 17,418 × *g* for 1 h at 5°C and the pellet was resuspended in 1/10 of the original volume in cold TN_50_ buffer (10 mM Tris [pH 7.5], 50 mM NaCl). The suspension was dialyzed overnight against TN_50_ buffer using a Slide-A-Lyzer dialysis cassette (Pierce) (3.5 molecular weight cutoff [MWCO]). The tailocin particles were sedimented at 90,619 × *g* for 2.5 h at 5°C using a Beckman type 60Ti rotor, the pellet was resuspended in P-buffer, and the titer for activity was determined as described above.

### Preparative isoelectric focusing.

For purposes of imaging and protein analysis, further purification was performed. Preparative isoelectric focusing (IEF) generated acceptably pure tailocin preparations. For IEF, 200 ml of an UV-induced tailocin lysate was concentrated by ultracentrifugation (90,619 × *g* for 2.5 h at 5°C) using a type 60Ti rotor in a Beckman L8-70 M ultracentrifuge. The pellet was resuspended in 2.5 ml sterile ultrapure water (Invitrogen) containing 0.3% (wt/vol) octylglucoside and 5% (vol/vol) glycerol. Three milliliters of Bio-Rad Ampholyte 3/10 was added to a buffer containing 3 M urea, 0.3% octylglucoside, and 0.3% (wt/vol) CHAPS **{**3-[(3-cholamidopropyl)-dimethylammonio]-1-propanesulfonate**}** to make a final volume of 50 ml. Anion exchange membranes were equilibrated in 0.1 M NaOH and cation exchange membranes in 0.1 M H_3_PO_4_. The tailocin preparation was applied to a Rotofor chamber (Bio-Rad) that had been prefocused at 15 W of constant power for 1 h. The chamber was run at 15 W and 4°C until the current stabilized. The pH of each fraction (∼20 fractions) was determined. The fractions were adjusted using 1 M Tris-HCl (pH 7.0) to a final concentration of 0.1 M, and the activity was measured by the spot-dilution method as described above.

### Electron microscopy.

An IEF-purified tailocin sample (2 × 10^11^ KU ml^–1^) diluted 1:5 in P-buffer was negatively stained for transmission electron microscopy (TEM) as described by Ahern et al. (53). Specimens were observed on a Jeol 1200EX TEM (Microscopy and Imaging Center, Texas A&M University) operating at an acceleration voltage of 100 kV. Images were recorded at calibrated magnifications by the use of a charge-coupled-device (CCD) camera, and measurements were acquired using Image J software.

### Effects of heat and enzyme treatments.

The temperature stability of tailocin BceTMilo was assessed by heating a large-scale purified tailocin preparation (10^10^ KU ml^–1^) to 50°C and 80°C for 60 min or to 100°C for 3 and 10 min. Samples were immediately chilled on ice after heating and assayed for activity by the spot dilution assay as described above.

The enzymatic stability of the tailocin with respect to trypsin (131,000 U mg^–1^), α-chymotrypsin (83.9 U mg^–1^), proteinase K (34 U mg^–1^), protease (4 U mg^–1^), lipase (20,000 U mg^–1^), papain (24 U mg^–1^), or lysozyme (70,000 U mg^–1^) was assayed using conditions previously described (C. F. Gonzalez and B. S. Kunka [56]). All enzymes were purchased from Sigma. Tailocin preparations (10^10^ KU ml^–1^) were incubated with each enzyme at a final concentration of 500 μg ml^–1^. Enzyme treatments performed with α-chymotrypsin and trypsin were performed at 25°C for 60 min, with all other enzyme/tailocin mixtures incubated at 37°C for 60 min. Tailocin activity, after incubation with individual enzymes, was assayed as described above.

### pH stability.

Tailocin prepared using the large-scale method was dialyzed against buffers (pH 2 to pH 10) using a Slide-A-Lyzer dialysis cassette (3.5 MWCO). The tailocin solution was dialyzed for 18 h with two exchanges against each of the following buffers: 0.05 M glycine hydrochloride buffer (pH 2.04), 0.05 M citrate buffer (pH 4.78), 0.05 M Tris-HCl buffer (pH 6.8 and 8.8), and 0.05 M carbonate-bicarbonate buffer (pH 10.64). The control was dialyzed against P-buffer. The contents were then serially diluted with P-buffer and assayed for activity as described above. All buffers were spotted onto overlays to confirm that the killing activity was not due to buffer activity.

### SDS-PAGE.

The major proteins of tailocin BceTMilo were separated using SDS-PAGE gel electrophoresis. An 8-μg volume of IEF-purified tailocin sample was added to 20 μl of SDS loading buffer (50 mM Tris-HCl [pH 7.5] with 0.01 g of SDS, 1.54 mg of dithiothreitol, and 0.1 mg ml^−1^ of bromophenol blue), heated at 95°C for 5 min, and applied to either a 7.5% or a 10 to 20% gradient Tris-HCl Mini-Protean gel (Bio-Rad, Hercules, CA). Electrophoresis was carried out at 200 V (constant) as described by Laemmli (57). Precision Plus protein standard (Bio-Rad/161-0374) was used as a standard. The gels were stained with Coomassie brilliant blue R250 (Bio-Rad).

### Liquid chromatography-tandem mass spectrometry (LC-MS/MS) analysis.

To conduct LC-MS/MS analysis of the tailocin major proteins, the 43-kDa and 17-kDa protein bands were excised from a 10 to 20% Tris-HCl gel, and the 100-kDa and 30-kDa bands were excised from a 7.5% SDS Tris-HCl gel. The proteins were subjected to LC-MS/MS analysis on a Quad-Time of Flight (Q-TOF) Premier mass spectrometer (University of Michigan Biomedical Core Facility—Proteomics and Peptide Synthesis Core). Protein LynX Global Server and Mascot search engines were used to search the Swiss-Prot database (http://ca.expasy.org/sprot) and the NCBI database (https://blast.ncbi.nlm.nih.gov/Blast.cgi) (58, 59).

### DNA manipulations.

Genomic DNA was extracted using a DNeasy kit (Qiagen, Valencia, CA), plasmid DNA was prepared using a Miniprep kit (Qiagen), and gel extractions and PCR product purification were conducted using a Qiaquick gel extraction kit and a Qiaquick PCR purification kit, respectively, according to the manufacturer's instructions (Qiagen). All restriction endonucleases, *Taq* DNA polymerase, and T4 DNA ligase were purchased from New England BioLabs (Ipswich, MA) and were used according to the manufacturer's instructions. Oligonucleotide primers were synthesized by Integrated DNA Technologies (Coralville, IA). DNA sequencing was performed at the Institute of Developmental and Molecular Biology Gene Technologies Laboratory at Texas A&M University.

### Sequencing assembly and annotation of BceTMilo genes.

To understand the genetic organization of BceTMilo, BC0425 genomic DNA was isolated using modifications of the cetyltrimethylammonium bromide (CTAB) method (60). BC0425 DNA was sequenced using 454 genome sequencing technology (Duke University, Institute for Genome Sciences & Policy). Sequence information was assembled into contigs using the Newbler sequence assembly program (Roche, USA). Genemark was used to predict all possible open reading frames. To annotate contig 77, which contains the BceTMilo genes, Genemark was first used to identify all possible ORFs (61). The Artemis Comparison Tool (Sanger Centre) was then used to identify the most likely start codon for each ORF by searching for the presence of a potential ribosomal binding site (RBS) (62). Each identified ORF was then compared to the NCBI protein database using BLASTP (https://blast.ncbi.nlm.nih.gov/Blast.cgi) to assign putative functions (58). InterProScan (http://www.ebi.ac.uk/Tools/webservices/services/interproscan), LipoP (http://www.cbs.dtu.dk/services/LipoP/), and TMHMM (http://www.cbs.dtu.dk/services/TMHMM/) were used to identify conserved domains, lipoprotein processing signals, and transmembrane domains (TMDs), respectively. Genome maps were drawn utilizing the programs DNA Master (http://cobamide2.bio.pitt.edu/computer.htm) and Inkscape (https://inkscape.org/en/).

### Lipopolysaccharide extraction.

Lipopolysaccharide (LPS) extracts from tailocin-sensitive and -resistant strains were obtained using the hot phenol method as described by Apicella et al. (63). The dry weight of purified LPS was determined, and the purified LPS was resuspended in endotoxin-free water (Sigma) to a concentration of 1 to 2 μg/ml.

### Electrophoretic analysis of LPS.

LPS samples were analyzed using a Laemmli SDS-gel electrophoresis system (57). LPS preparations were dissociated in SDS loading buffer and heated at 90 to 100°C for 5 min. LPS was resolved by running 1.0 μg of each extract on a 16.5% Tris-Tricine SDS mini-gel (Bio-Rad) for 6 h at 60 V and visualized by silver staining (64).

### LPS adsorption assay.

A LPS adsorption assay was performed using a purified tailocin preparation (10^11^ KU ml^–1^). The tailocin preparation was diluted (1:10) with P-buffer amended with 5 mM CaCl_2_ (PC-buffer) and mixed with increasing amounts of LPS preparations. After incubation at 37°C for 30 min, the mixtures were assayed for activity by spotting a 1:2 dilution series on overlays seeded with B. cenocepacia strain PC184, as described above.

### Sugar inhibition assay.

The sugar inhibition assay was performed as described by Dawes (65) with minor modifications. The effects of d-galactose, α-d-glucose, d-fructose, d-mannoheptose, l-rhamnose, d-cellobiose, lactose, sucrose, and d-raffinose on the adsorption of tailocin BceTMilo to strain PC184 were tested. Strain PC184 was grown in 1 liter TNB (at 200 rpm) to an *A*_600_ = 1. Cells were centrifuged (10,000 × *g*) at 4°C for 5 min. The cells were washed in 40 ml P-buffer, centrifuged, and then resuspended in 4 ml P-buffer. Two ml of the bacterial suspension (10^11^ cells ml^–1^) was mixed with 500 μl of tailocin BceTMilo (10^9^ KU ml^–1^) and 2.5 ml of 1.2 M sugar (final concentration of 0.6 M). The mixture was incubated at 37°C, and 500-μl samples were taken at 0, 1, 3, 6, and 9 min postmixing. Samples were immediately centrifuged, and supernatants were filter sterilized. The remaining tailocin activity of the samples was determined using a spot assay. The percentage of unadsorbed tailocins was calculated by dividing the titer of each sample by the titer of the buffer control.

### Construction of deletion mutants in B. cenocepacia BC0425 and K56-2.

Deletion of *recA* in BceTMilo producer strain BC0425 and deletion of *waaL* and *waaC* in indicator strain K56-2 were performed using the system developed by Hamad et al. (51), except that the constructed suicide vectors (pMo130::*recA*-UD, pMo130::*waaL*-UD, and pMo130::*waaC*-UD) were introduced into B. cenocepacia strain BC0425 or K56-2 by conjugation using triparental mating with pRK2013 as the mobilizing plasmid. In brief, donor, mobilizer, and recipient suspensions were made in LB broth from cultures grown on solid media under selective conditions as appropriate for 18 h. Bacterial suspensions were adjusted spectrophotometrically to *A*_600_ = 0.5, mixed at equal ratios (1:1:1), and transferred to a positively charged sterile membrane layered on a 100-mm-by-15-mm LB agar petri dish. Following 18 h of incubation at 37°C, the cells from the mating mixtures and respective controls were washed twice in phosphate buffer (0.125 M, pH 7.1) by centrifugation (12,096 × *g* for 10 min at 5°C). The bacterial pellets were resuspended in phosphate buffer, and dilutions were plated on LB agar plates amended with 600 μg ml^−1^ Km and 20 μg ml^−1^ Tc for selection of single-crossover events. PCR primers for regions flanking *recA* were designed based on the annotated sequence of BC0425. Since B. cenocepacia strain K56-2 is clonally related to the sequenced strain, J2315 (33, 66), PCR primers for regions flanking *waaL* and *waaC* in strain K56-2 were designed based on J2315 sequences BCAL2404 to BCAL2406 and BCAL3111 to BCAL3113, respectively (www.sanger.ac.uk/Projects/B_cenocepacia/). Primers used for constructing the suicide vectors (pMo130::*recA*-UD, pMo130::*waaL*-UD, and pMo130::*waaC*-UD) are listed in Table 4. The deletions of the respective genes were confirmed by PCR.

**TABLE 4:** Primers used in this study

Primer	Sequence^*a*^
*recA*-up-F	5′-GTGGCTAGCTGACCGGATGGATCTGGCGCG-3′
*recA*-up-R	5′-CTCAGATCTCAGCGTCTGTTGGAGGCGCGC-3′
*recA*-down-F	5′-GTGAGATCTGAGTGATGGTTGCGCGCCGAG-3′
*recA*-down-R	5′-GTGAAGCTTACGGGCTCGGATAGCGGCATG-3′
*waaL*-up-F	5′-GTGCGGCCGCTCGCTTCGCCGAGCACCATG-3′
*waaL*-up-R	5′-GTGGGATCCTCTGGGCATTTGCCGGGCTGG-3′
*waaL*-down-F	5′-GTCGGATCCAAGACGCTCCATACGCGCCG-3′
*waaL*-down-R	5′-GACTTCTAGAGCGCACCATCTCCTGCTCGG-3′
*waaC*-up-F	5′-CTGGCTAGCCCCGGGTATTGCGTCGAA-3′
*waaC*-up-R	5′-GTCAGATCTCCTACTGGTCGCCGAACGTCGT-3′
*waaC*-down-F	5′-GTCAGATCTCAGCCGGCGACAGACATAAAG-3′
*waaC*-down-R	5′-CGTAAGCTTCAATCGACGTCGCGGATCAGT-3′
*recA*-comp-F	5′-CTCGGATCCGGAAGATAGCAAGAAGGGCTC-3′
*recA*-comp-R	5′-CTCTCTAGACTCGGCGCGCAACCATCACTC-3′
*waa*L-comp-F	5′-GTGGATCCCATGAGCGGGCTGTCGGTGG-3′
*waaL*-comp-R	5′-CACTTCTAGACAGCAATCGCACGGGCTTGC-3′
*waaC*-comp-F	5′-CACCTGCAGTCGGTTCGCGTGTGGACAGC-3′
*waaC*-comp-R	5′-GACGGATCCTATGTCTGTCGCCGGCTGCC-3′

a Added restriction sites are underlined.

### In-*trans* complementation of BC0425 and K56-2 mutants.

Plasmids pMo168::*recA*-comp, pMo168::*waaL*-comp, and pMo168::*waaC*-comp were constructed as replicative vectors for in-*trans* complementation of the BC0425 Δ*recA*, K56-2 Δ*waaL*, and K56-2Δ *waaC* mutants, respectively. Plasmids were introduced into the deletion mutants by triparental mating as described above. Primers used for amplifying the wild-type *recA*, *waaL*, and *waaC* genes are listed in Table 4. The presence of the wild-type *recA*, *waaL*, and *waaC* genes was confirmed by PCR.

### Accession number(s).

In accordance with Texas A&M Center for Phage Technology policy (https://cpt.tamu.edu), the tailocin from B. cenocepacia BC0425 was designated BceTMilo. The genome was deposited in GenBank under accession number KY316063.

## Supplementary Material

Supplemental material
