# Supplementary material for: A Broad-Host-Range Tailocin from Burkholderia cenocepacia
Source: Appl Environ Microbiol. 2017 May 1;83(10):e03414-16. doi: 10.1128/AEM.03414-16 (PMC5411513; doi:10.1128/AEM.03414-16)
Supplement: Supplemental material [file supp_83_10_e03414-16__index.html]

Supplemental material 

# A Broad-Host-Range Tailocin from Burkholderia cenocepacia

## Supplemental material

- Supplemental file 1 -

  Sequence alignments of LC-MS/MS identified peptides of BceTMilo with homologous protein subunits (Fig. S1); *Burkholderia* screening panel (Table S1); susceptibility of *Burkholderia* species to BceTMilo or pyocin R, R2, or R5 (Table S2).

  PDF, 68K
- Supplemental file 2 -

  Tailocin BceTMilo annotation table (Table S3).

  XLSX, 15K
