# Supplementary material for: A Broad-Host-Range Tailocin from Burkholderia cenocepacia
Source: Appl Environ Microbiol. 2017 May 1;83(10):e03414-16. doi: 10.1128/AEM.03414-16 (PMC5411513; doi:10.1128/AEM.03414-16)
Supplement: Supplemental material [file AEM.03414-16_zam999117820s1.pdf]

A

BceTMilo (17 kDa)	35	MEDYQGGGMSGPIKVDFGQEGIQLE	59
B. cenocepacia (J2315) phage major tail tube	35	MEDYQGGGMSGPIKVDFGQEGIQLE	59
B. ubonesis phage major tail tube	33	MEDYQGGGMSGPIKVDFGQEGIQLE	57

B

BceTMilo (30 kDa)	36	TLDAPPARTPTTWPFYRNDVWTLLP	60
B. cenocepacia (J2315) hypothetical protein	36	TLDAPPARTPTTWPFYRNDVWTLLP	60
B. pseudomallei tail fiber assembly	36	TLDAPPARTPTTWPFYRDGAWFLLP	60

C

BceTMilo (43 kDa)	36	ADADASAFPLNTPVLLTNVVAALGK	60
B. pseudomallei phage major tail sheath	36	ADADASAFPLNTPVLLTNVVAALGK	60
B. cenocepacia (J2315) phage major tail sheath	36	SDADATAFPLDTPVLLTNVVAALGK	60
Phage P2 major tail sheath	36	SDADAETFPLNKPVLITNVQSAISK	60

D

BceTMilo (100 kDa)	56	SFGGANVAPDTIHTTLK	72
B. cenocepacia (J2315) phage tail protein	56	SFGGANVAPDTIHTTLK	72
B. pseudomallei phage-related tail protein	56	TFGGENVAPDTVHVVIQ	72

FIG S1 Sequence alignments of LC-MS/MS identified peptides of BceTMilo with homologous protein subunits. (A) Peptide sequence from 17 kDa subunit of BceTMilo; phage tail tube, *B. cenocepacia* J2315 (BCAL0094); phage tail tube, *B. ubonesis* (WP_010095894.1). (B) Peptide sequence from 30 kDa subunit of BceTMilo; hypothetical protein, *B. cenocepacia* J2315 (BCAL0096); caudovirales tail fiber assembly, *B. pseudomallei* (WP_044368048.1). (C) Peptide sequence from 43 kDa subunit of BceTMilo; phage major tail sheath, *B. pseudomallei* K96243 (YP_106778.1); putative phage major tail sheath, *B. cenocepacia* J2315 (BCAL0095); major tail sheath, phage P2 (NC_001895.1). (D) Peptide sequence from 100 kDa subunit of BceTMilo; phage tail fiber, *B. cenocepacia* J2315 (BCAL0097); phage tail, *B. pseudomallei* (WP_080399442.1). Amino acid residues exhibiting identity are shaded.

TABLE S1 *Burkholderia* screening panel

Strain	Species and/or lineage	Reference or source
Hines #1	*Ralstonia pickettii*[a]	1
ATCC25416	*B. cepacia*	1
AU0007	*B. cenocepacia* ET12[b]	BcRLR[c]
AU2294	*B. cenocepacia*	BcRLR
AU2433	*B. cenocepacia*	BcRLR
BC0425	*B. cenocepacia*	BcRLR
AU0493	*B. cenocepacia*	BcRLR
AU2225	*B. cenocepacia*	BcRLR
HI2850	*B. cenocepacia* ET12	BcRLR
AU0644	*B. cenocepacia* MW[b]	BcRLR
AU0918	*B. cenocepacia*	BcRLR
AU2589	*B. cenocepacia* MW	BcRLR
AU1547	*B. cenocepacia*	BcRLR
AU2079	*B. cenocepacia* PHDC[b]	BcRLR
AU2027	*B. cenocepacia*	BcRLR
AU1054	*B. cenocepacia* PHDC	BcRLR
AU2622	*B. cenocepacia*	BcRLR
AU0137	*B. cenocepacia* PHDC	BcRLR
AU0583	*B. cenocepacia*	BcRLR
AU0202	*B. cenocepacia* PHDC	BcRLR

[a] Formerly known as *Burkholderia pickettii*
[b] ET12 lineage, Midwest(MW) and PHDC clones defined in reference 2
[c] *Burkholderia cepacia* Reference Laboratory and Repository

References

1. Gonzalez CF, Vidaver AK. 1979. Bacteriocin, plasmid and pectolytic diversity in *Pseudomonas cepacia* of clinical and plant origin. J Gen Microbiol 110:161–170. https://doi.org/10.1099/00221287-110-1-161.

2. LiPuma JJ. 2010. The changing microbial epidemiology in cystic fibrosis. Clin Microbiol Rev 23:299–323. https://doi.org/10.1128/CMR.00068-09.

TABLE S2 Susceptibility of *Burkholderia* species to BceTMilo or pyocin R, R2 or R5

Burkholderia species	No. Susceptible BceTMilo /No. Tested	[a]No. Susceptible pyocin R1 /No. Tested	[a]No. Susceptible Pyocin R2 /No. Tested	[a]No. Susceptible Pyocin R5 /No. Tested
B. cepacia	3/3	0/3	0/3	0/3
B. multivorans	9/11	0/3	0/3	0/3
B. cenocepacia	24/41	0/22	1/22	3/22
B. stabilis	2/2	0/2	0/2	0/2
B. vietnamiensis	3/3	0/2	0/2	1/2
B. dolosa	2/3	0/3	0/3	0/3
B. ambifaria	3/3	0/3	0/3	0/3
B. anthina	1/3	0/3	0/3	0/3
B. pyrrocinia	3/3	0/3	0/3	0/3
B. contaminans	0/1	ND	ND	ND
B. gladioli (Non-Bcc)	3/3	0/3	0/3	0/3
B. glumae (Non-Bcc)	23/25	0/0	0/0	0/0
Total Susceptible/ Total Tested	76/101	0/47	1/47	4/47

All isolates were tested by both broth microdilution and spot assay using 1:10 serial dilutions of tailocin (range from 1/10 to 1/100,000 dilutions). Isolates were considered susceptible when the spot assay revealed at least a partially clear spot at the first dilution of tailocin, and had an MIC of ≤1/10 using a stock of 5×10^9 KU/ml. Resistant isolates showed no activity at the 1/10 spot and an MIC>1/10. MICs ranged from >1/10 to 1/10,000. *B. gladioli* and *B. glumae* are not members of the Bcc.

[a] A subset of *Burkholderia* isolates were tested for sensitivity to pyocins R1, R2, or R5 using spot test protocol.
